# Perception of Harmfulness of Various Tobacco Products and E-Cigarettes in Poland: A Nationwide Cross-Sectional Survey

**DOI:** 10.3390/ijerph18168793

**Published:** 2021-08-20

**Authors:** Mateusz Jankowski, Iwona Wrześniewska-Wal, Aurelia Ostrowska, Aleksandra Lusawa, Waldemar Wierzba, Jarosław Pinkas

**Affiliations:** 1 School of Public Health, Centre of Postgraduate Medical Education, 01-826 Warsaw, Poland; iwrzesniewska@cmkp.edu.pl (I.W.-W.); aurostro@interia.pl (A.O.); alusawa@cmkp.edu.pl (A.L.); jpinkas@cmkp.edu.pl (J.P.); 2 UHE Satellite Campus in Warsaw, University of Humanities and Economics in Łódź, 01-513 Warsaw, Poland; waldemar.wierzba@cskmswia.pl; 3 Central Clinical Hospital of the Ministry of the Interior and Administration in Warsaw, 02-507 Warsaw, Poland

**Keywords:** tobacco, harm perception, tobacco control, public health, smoking, Poland

## Abstract

Perceptions of the harmfulness of tobacco products may be a determinant of smoking behaviors. This study aimed to: (1) assess the perception of harmfulness of various tobacco products and e-cigarettes in Poland as well as (2) to assess the awareness of the health effects of using tobacco and e-cigarettes. A cross-sectional survey was conducted in 2019 with a nationally representative sample of 1011 individuals aged 15 and over. In the studied group, 22.3% were smokers. Smokeless tobacco was most likely to be perceived as less harmful than cigarettes (25%), followed by water pipe (24.5%), heated tobacco products (22%), e-cigarettes (21.6%), slim cigarettes (17.1%), flavored cigarettes (except menthol ones) (16.1%), menthol cigarettes (15.6%) and cigarillos (12.6%). In this study, 10% of respondents denied that smoking causes serious diseases. Most of the respondents (88.9%) were aware that smoking causes lung cancer (88.9%), but only 70.4% were aware that smoking causes stroke. Smokers compared to non-smokers were less likely to declare that smoking causes a stroke (OR: 0.43, 95%CI: 0.31–0.59; *p* < 0.001) or myocardial infarction (OR: 0.41, 95%CI: 0.29–0.60; *p* < 0.001). There were no significant differences (*p* > 0.05) in the perception of harmfulness of various tobacco products and e-cigarettes by gender, age, or occupational status.

## 1. Introduction

Combusted tobacco use is the leading cause of preventable death and disability worldwide [1,2]. Smoking harms nearly every organ in the body [3]. It is estimated that smoking causes one of every three deaths from cardiovascular disease and 3 out of every 10 cancer deaths [3,4]. Smokers are more likely than nonsmokers to develop coronary heart disease (by 2 to 4 times), stroke (by 2 to 4 times), and lung cancer (by 25 times) [3,4]. The global economic cost of tobacco-related diseases is equivalent to 1.8% of the world’s annual gross domestic product [5].

According to World Health Organization (WHO) estimates, 1.3 billion people globally use tobacco products [2]. Among the WHO regions, Europe has the highest prevalence of tobacco use among adults [6,7]. In 2017, in the European Union (EU) over a quarter (26%) of people aged 15 and over were smokers, wherein the highest prevalence of smoking was in Greece (37%), Bulgaria (36%), and France (36%) in contrary to Sweden where only 7% of inhabitants were smokers [6]. The most popular tobacco products among smokers were boxed cigarettes (70%) and hand-rolled cigarettes (23%) [6]. Cigars, cigarillos, and pipes were used by less than 1% of the EU population [6]. Between 2006 and 2017 the prevalence of tobacco use in the EU has decreased by six percentage points [6]. This significant public health achievement is mainly related to an increased awareness of the health effects of tobacco use as well as more restrictive national tobacco control policies [7,8].

In recent years, novel nicotine and tobacco products such as electronic cigarettes (e-cigarettes) and heated tobacco products (HTPs) are gaining popularity [9]. These products are advertised by the tobacco industry as being less harmful than traditional cigarettes [9,10]. However, the long-term health effects of e-cigarette or HTPs use are unknown [9]. Some studies demonstrated lower emissions of toxicant substances during e-cigarette or HTPs use, but clinical studies suggest that the use of these products may still increase the risk of cardiovascular diseases such as myocardial infarction or stroke [11,12,13,14]. These products are perceived as less harmful by society, with significant differences by occupation, gender, or age [15,16].

Poland is the sixth EU country in terms of the frequency of tobacco use. However, in the past decade, substantial progress in tobacco control was observed. In Poland, the prevalence of adult daily smoking (aged 15 and over) decreased from 31% in 2011 to 21% in 2019 [17]. This change in attitudes towards tobacco use in Poland may result from the strengthening of the anti-tobacco policy in 2010 with the adoption of smoking bans in public places, restrictions on advertising and marketing, text health warnings, and other broad-based actions [18]. Moreover, in 2012/2013, cytisine—a low-cost medication for smoking cessation was switched to the over-the-counter (OCT) category [19]. Tutka et al. showed that cytisine’s increased use has been posited as an important factor responsible for reduced smoking rates in Poland (with a continuous upward trend of use among those who attempt to quit smoking) [19].

There are multiple tobacco products available in Poland, including boxed cigarettes, hand-rolling tobacco, menthol cigarettes (up to 20 May 2020), slim cigarettes, cigars, cigarillos, water pipes (shisha), as well as smokeless tobacco (e.g., snus, chewing tobacco). In 2006, e-cigarettes were introduced to the Polish market and HTPs are available from 2017 [9]. According to the tobacco control Act in Poland, all products that meet the criteria of tobacco products (including cigarettes, cigars, cigarillos, water pipe, smokeless tobacco), novel tobacco products (including HTPs), and e-cigarettes are subject to the following regulations: minimum age of sales, tobacco packaging and labeling including required health warnings, tobacco products ingredients reporting, ban on tobacco and e-cigarette use in public places, ban on tobacco advertising, promotion, and sponsorship [20]. The same rules are applied to both combustible and non-combustible tobacco products.

Regular monitoring of public perceptions about tobacco products and e-cigarettes can help to inform the public health authorities as it develops tobacco control policies and regulations for various tobacco products and e-cigarettes [21]. Perceptions of harmfulness may be a determinant of tobacco products initiation, tobacco products selection, smoking habits, and quit attempts. Currently, there is little research on the perception of the harmfulness of various tobacco products and e-cigarettes in Poland, and most of them are limited to specific populations (adolescents, medical students, social care beneficiaries) [22,23,24]. There is a lack of up-to-date data on the perception of the harmfulness of various tobacco products and e-cigarettes in the general population in Poland.

Therefore, the objectives of this study were to: (1) to assess the perception of harmfulness of various tobacco products and e-cigarettes in Poland as well as (2) to assess the awareness of the health effects of using tobacco and e-cigarettes.

## 2. Materials and Methods

### 2.1. Study Desing and Population

This cross-sectional study was carried out in September 2019 on a nationwide, representative sample of 1011 inhabitants of Poland aged 15 years and over. The computer-assisted web interview (CAWI) technique was used [25]. Address-based sampling was applied with a stratification model that includes gender, age, size of domicile, and the territorial distribution within administrative regions (voivodeships) in Poland. A random quota sample was selected from the National Official Register of the Territorial Division of the Country (TERYT) sampling frame. Moreover, the stratification was based on demographic data from the “Population Report. Status and structure in territorial division” [17].

All the interviews were carried out by a specialized survey company—Kantar—on behalf of the Chief Sanitary Inspectorate and the research team, which provides the context of this research.

Participation in the study was voluntary and anonymous. The study protocol was approved by the Ethical Review Board at the Centre of Postgraduate Medical Education, Warsaw, Poland (consent number 51/PB/2020).

Participants were divided into two groups (smokers vs. non-smokers) according to their self-declared smoking status.

### 2.2. Questionnaire and Study Measures

The research tool was a questionnaire developed for the purpose of this study. In preparation for the questionnaire, we analyzed the previously published nationwide cross-sectional surveys on tobacco use as well as on attitudes towards various tobacco products [6], with particular emphasis on the Global Adult Tobacco Survey (GATS) [26]. The questionnaire included 42 questions related to attitudes and behaviors towards tobacco use. Questions also addressed personal characteristics.

Smoking status (tobacco use): Respondents were asked about their smoking status, using the questions: “Have you smoked at least 100 cigarettes (or a similar amount of other tobacco products) in your lifetime?” and “Do you currently smoke?”. Smokers were respondents who reported having smoked at least 100 cigarettes (or a similar amount of other tobacco products) during their lifetime and who reported current tobacco use (last 30 days). Respondents who reported having smoked fewer than 100 cigarettes (or a similar amount of other tobacco products) during their lifetime and/or do not currently smoke were classified as non-smokers.

In this study, we also asked the respondents about the use of e-cigarettes using the following question: “Do you currently use an e-cigarette?”. However, due to the low prevalence of e-cigarette use in a general population in Poland, we identified only 14 e-cigarette users. Out of all e-cigarette users, 4 were dual users. Due to the low number of exclusive e-cigarette users (*n* = 10), we do not prepare a separate analysis for e-cigarette users (the population number was too low to run separate analysis free from risk of serious bias).

Awareness of the health effects of using tobacco and e-cigarettes: Respondents were asked about their awareness of health effects of using tobacco and e-cigarettes, using the question: “Do you think that smoking causes serious diseases?” and “Do you think that smoking causes (1) stroke, (2) myocardial infarction, (3) lung cancer?” with three possible answers: “Yes”, “No”, “I do not know”. The same set of questions was asked for the perception of e-cigarettes. Respondents were also asked about their perception of the harmful effect of passive smoking on their health, with four possible answers: “I am very concerned”, “I am a little concerned”, “I am not very concerned”, “I am completely not concerned”.

Harm perceptions of various tobacco products and e-cigarettes: Respondents were asked about their perception of harmfulness of various tobacco products and e-cigarettes status, using the question: “Compared to traditional cigarettes, how harmful do you think (1) menthol cigarettes, (2) flavored cigarettes (except menthol), (3) slim cigarettes, (4) cigarillos, (5) smokeless tobacco, (6) water pipe (shisha), (7) electronic cigarettes (e-cigarettes) and (8) heated tobacco products?” with three possible answers: less harmful, just as harmful, more harmful.

Sociodemographics: Questions related to sociodemographic data included: gender (male/female), age (years), educational level (primary, vocational, secondary, or higher), occupational status, financial situation, and place of residence (rural/urban). Active occupational status included employees and self-employed. Passive occupational status included unemployed, students, pensioners, and retirees. The financial situation was assessed with the question: “How do you assess your own/your family’s financial situation? (high, medium, low)” (this question was chosen due to the numerous missing data in the case of the question about the amount of monthly income).

### 2.3. Statistical Analysis

The data were analyzed with SPSS version 28 (IBM, Armonk, NY, USA). Demographic weighting was applied (age, gender and geographical location). The distribution of categorical variables was shown by proportions along with 95% confidence intervals (95%CI). Statistical testing to compare categorical variables was completed using the independent samples chi-square test. Data were presented separately for smokers and non-smokers.

Associations between sociodemographic factors (gender, age, educational level, occupational status, financial situation, place of residence, smoking status) and awareness of selected health effects of using tobacco (smoking as a risk factor for stroke or myocardial infarction) and e-cigarettes (e-cigarette use as a cause of serious diseases) were analyzed using the logistic regression analyses. Smoking as a risk factor for stroke, smoking as a risk factor for myocardial infarction, e-cigarette use as a cause of serious diseases were considered separately as a dependent variable in the model. These variables were selected because the harmful effect of smoking on the risk of myocardial infarction and stroke is well described in the literature. Moreover, currently available data confirm that e-cigarette use causes serious diseases. However, because currently there is a lack of prospective cohort studies on the impact of e-cigarette use on the risk of myocardial infarction, stroke, or lung cancer we cannot identify this relationship, so we did not include these questions as variables in the multivariate analysis.

The sociodemographic characteristics (gender, age, educational level, occupational status, financial situation, place of residence, smoking status) were considered as independent variables. In univariate logistic regression analyses, all variables were considered separately. Multivariate logistic regression analyses included all sociodemographic variables. The strength of association was measured by the odds ratio (OR) and 95% confidence intervals (CI). Statistical inference was based on the criterion *p* < 0.05.

## 3. Results

### 3.1. Characteristics of the Study Population

The analysis is based on responses to survey forms received from 1011 individuals (52.1% females) aged 15+ years, 22.3% were smokers. The characteristics of the study population have been published previously [17].

### 3.2. Awareness of the Health Effects of Using Tobacco and E-Cigarettes

Most of the respondents (83.6%) admitted that smoking causes serious diseases (without differences between smokers and non-smokers (*p* = 0.1)). Out of three different tobacco-related diseases analyzed in this study, most of the respondents (88.9%) were aware that smoking causes lung cancer (88.9%). The proportion of respondents who were aware that smoking causes cardiovascular diseases such as stroke or myocardial infarction was higher among non-smokers compared to smokers (74.4% vs. 56.5% and 84.5% vs. 70.8%, *p* < 0.001 respectively). Among the respondents, 57.9% declared, that e-cigarette use causes serious diseases, with significant differences between smokers and non-smokers (60.4% vs. 49.1%, *p* = 0.01). Over a quarter of respondents (27.9%) did not know whether e-cigarette use causes disease. Lung cancer was the most common (65.8%) health effect of e-cigarette use indicated by the respondents. More than half of respondents believed that e-cigarette use causes stroke (54.4%) or myocardial infarction (59.4%), wherein non-smokers compared to smokers (*p* < 0.001) more often declared that e-cigarette use causes cardiovascular diseases. Over half of the respondents (52.4%) were not concerned (not very concerned or completely not concerned) about the harmful effect of passive smoking on their health. Details are presented in Table 1.

There were no significant differences (*p* > 0.05) in awareness of the health effects of using tobacco and e-cigarettes by gender, age, or occupational status. Those who lived in urban areas more often declared that smoking causes serious diseases (85.9%) compared to those who lived in rural areas (79.7%; *p* = 0.03). There was no impact of place of residence on awareness of the health effects (cardiovascular diseases and lung cancer) of using tobacco and e-cigarettes. Respondents who had higher education (91.9%) more often declared that smoking causes serious diseases compared to those with primary (82.4%) or vocational education (78.0%; *p* = 0.002). Respondents with higher (72.1%) or secondary (72.9%) education more often declared that smoking causes stroke, compared to those with primary (68.8%) or vocational (67.1%; *p* = 0.03) education. Moreover, respondents who had higher education (94.6%) more often declared that smoking causes lung cancer compared to those with vocational education (82.8%; *p* = 0.004). A similar impact of educational level on awareness of the health effects was observed in the case of e-cigarettes. Those with higher education more often declared that e-cigarette use causes serious diseases, stroke, or lung cancer compared to those without higher education (*p* < 0.05).

### 3.3. Harm Perceptions of Various Tobacco Products and E-Cigarettes

Menthol cigarettes, other flavored cigarettes, and slim cigarettes were perceived as harmful as traditional cigarettes by 80% of respondents. Smokers compared to non-smokers more often declared that menthol cigarettes are less harmful than traditional cigarettes (19.9% vs. 14.4%; *p* = 0.01). There were no significant differences in perception of flavored cigarettes (except menthol), slim cigarettes, and cigarillos between smokers and non-smokers (*p* > 0.05). One-fourth of the respondents declared that smokeless tobacco is less harmful than traditional cigarettes with significant differences between smokers (32.3%) and non-smokers (22.9%; *p* = 0.01). A similar percentage of respondents (24.5%) declared that water pipe (shisha) is less harmful than traditional cigarettes. Among the respondents, 70% declared that e-cigarettes are as harmful as traditional cigarettes, wherein smokers compared to non-smokers more often declared that e-cigarettes are less harmful than traditional cigarettes (28.6% vs. 19.5%; *p* = 0.01). There were no statistically significant differences (*p* > 0.05) between smokers and non-smokers in the perception of the harmfulness of heated tobacco products. Details are presented in Table 2.

Those who lived in rural areas compared to those who lived in urban areas, more often declared that menthol cigarettes are less harmful than traditional cigarettes (18.6% vs. 13.8%; *p* = 0.04). Respondents who declared good financial situation more often indicated that menthol cigarettes are as harmful as traditional cigarettes (84.9%), compared to those with moderate (79.0%) or bad (79.1%) financial situation (*p* = 0.001). Moreover, respondents who declared good financial situation more often indicated that cigarillos are as harmful as traditional cigarettes (87.1%), compared to those with moderate (82.1%) or bad (81.6%) financial situation (*p* = 0.005). Respondents aged 30–39 years (33.8%) compared to those aged 50–59 (19.2%) or over 60 years (17.4%) more often declared that water pipe (shisha) is less harmful than traditional cigarettes (*p* = 0.01). There were no significant differences (*p* > 0.05) in the perception of harmfulness of other tobacco products (6 various products) and e-cigarettes by age. Moreover, there were no significant differences (*p* > 0.05) in the perception of the harmfulness of various tobacco products and e-cigarettes by gender, educational level, and occupational status.

### 3.4. Associates of Harm Perceptions of Various Tobacco Products and E-Cigarettes

The results of the univariate and multivariate regression analyses are presented in Table 3. Smokers compared to non-smokers were less likely to declare that smoking causes stroke (OR:0.43, 95%CI: 0.31–0.59; *p* < 0.001) and myocardial infarction (OR:0.41, 95%CI: 0.29–0.60; *p* < 0.001). Moreover, smokers compared to non-smokers were less likely to declare that e-cigarette use causes serious diseases (OR:0.63, 95%CI: 0.46–0.86; *p* < 0.01). In the multivariate logistic regression model, there was no influence (*p* > 0.05) of gender, age, educational level, occupational status, financial situation, and place of residence on the respondents’ awareness of smoking as a risk factor for stroke. Moreover, in the multivariate logistic regression model, there was no influence (*p* > 0.05) of gender, age, educational level, occupational status, financial situation, and place of residence on the respondents’ attitudes towards the perception of harmfulness of e-cigarette use.

The youngest respondents (15–19 years old) were almost three times less likely (OR:0.35, 95%CI: 0.17–0.70; *p* < 0.01) to declare that smoking causes myocardial infarction compared to respondents aged 60 years and over. Respondents with a bad financial situation were almost twice less likely (OR:0.48, 95%CI: 0.28–0.81; *p* < 0.01) to declare that smoking causes myocardial infarction compared to respondents with a good financial situation. Details are presented in Table 3.

## 4. Discussion

To the authors’ best knowledge, this is the most up-to-date study on the perception of the harmfulness of various tobacco products and e-cigarettes in Poland. This study showed that among inhabitants of Poland aged 15 years and over, non-combustible tobacco products are perceived as less harmful than combustible tobacco products. Novel nicotine-containing products such as e-cigarettes and HTPs were also perceived as less harmful than traditional cigarettes. While smoking was perceived as a risk factor for serious diseases by most of the respondents, slightly more than half of the respondents admitted that e-cigarette use may cause serious diseases. Moreover, over half of the respondents declared that they were not concerned about the harmful effect of passive smoking on their health. In this study, most of the respondents were aware that smoking causes lung cancer, but significant gaps were identified in the awareness of the cardiovascular effect of smoking—nearly 20% of respondents did not indicate myocardial infarction and 30% did not indicate a stroke as a tobacco-related disease. Current tobacco use was the most important factor associated with the perception of the harmfulness of various tobacco products and e-cigarettes. Moreover, attitudes towards the perception of the harmfulness of tobacco and e-cigarettes differed by educational level and financial situation.

According to the WHO statement, all forms of tobacco are harmful, and there is no safe level of exposure to tobacco [2]. However, numerous researches have showed that non-combustible tobacco products are considered less harmful than combustible tobacco products by the general public [16,21]. The lack of characteristics smelling may lead to misperceptions of the health effects of exposure to tobacco products. Moreover, the marketing strategies of the tobacco industry also influenced the perceptions of different tobacco products. For example, flavored cigarettes and menthol cigarettes were targeted to the youngest age group and female smokers due to the reduction of unpleasant odors during smoking [27]. Perception of the harmfulness of various tobacco products may also result from the health literacy competencies, educational level, and socioeconomic status [15,16,22,23].

This study showed that non-combustible tobacco products were perceived as less harmful than combustible tobacco products which are in line with previous studies [4,6]. Novel nicotine-containing products such as e-cigarettes and HTPs were also perceived as less harmful than traditional tobacco, which may lead from the fact that these products were marketed as a “reduced-risk alternative to traditional cigarettes” [9,10]. Perceptions of the harmfulness of various tobacco products and e-cigarettes relative to cigarettes varied widely across the eight products analyzed in this study. Smokeless tobacco was most likely to be perceived as less harmful than cigarettes (25%), followed by water pipe (24.5%), HTPs (22%), e-cigarettes (21.6%), slim cigarettes (17.1%), flavored cigarettes (except menthol ones) (16.1%), menthol cigarettes (15.6%), and cigarillos (12.6%). There were statistically significant differences in perception of four out of eight products analyzed in this study. There were no significant differences in perception of harmfulness of all analyzed tobacco products and e-cigarettes by gender, age (except the water pipe), educational level, and occupational status. We can suspect that the tobacco industry’s marketing efforts on the health effect of tobacco use are targeted strictly to smokers to discourage them from quitting smoking. Moreover, a relatively high percentage of respondents (>20%), who declared that e-cigarettes and HTPs are less harmful than traditional cigarettes may result from the fact that these products are aggressively marketed in the EU [28].

In this study, 10% of respondents denied that smoking causes serious diseases. We also observed a significant gap in knowledge on the cardiovascular effect of tobacco use. There is strong scientific evidence proving that smoking more than doubles the risk of myocardial infarction or stroke [2,3,4]. In this study, nearly 20% of respondents did not indicate myocardial infarction and 30% did not indicate a stroke as a tobacco-related disease. Lung cancer was the most frequently indicated tobacco-related disease. This observation may result from the belief that, due to the inhalation of smoke into the lungs, the harmful effects of tobacco smoking are most often observed in this organ. Educational campaigns on the harmful effects of smoking in Poland, for the most part, were carried out by respiratory physicians and public health specialists and included communication on lung cancer and COPD. Cardiovascular diseases are the leading cause of death in Poland [29]. The lack of awareness of the impact of smoking on the risk of cardiovascular disease indicates an urgent need for educational measures.

This study showed that more than half of respondents were not concerned about the harmful effect of passive smoking on their health. It is estimated that only two-thirds of the Polish population has adopted a total smoke-free home rule [30]. Despite the implementation of the smoking ban in most public places, 11.7% of adults in Poland are exposed to secondhand smoke in public transport stops and facilities, and 6.7% are exposed to secondhand smoke in the workplace [30]. The lack of concerns about the harmful effect of passive smoking on health declared by more than half of respondents may result from the low awareness of the health effect of passive smoking. This observation suggests that there is a need to strengthen tobacco control policy (e.g., ban smoking in cars with children, stricter penalties for smoking in public places, promotion of smoke-free places) and increase public awareness of the health effects of secondhand smoke exposure [31].

There are few papers on the perception of the harmfulness of various tobacco products and e-cigarettes in Poland, and most of them are limited to specific populations. In the study by Milcarz et al. carried out among 1817 social care beneficiaries in Poland, men compared to women, consider slim cigarettes, smokeless tobacco, and e-cigarettes to be more harmful than traditional cigarettes (*p* < 0.05) [22]. In our study, there were no significant differences in the perception of the harmfulness of these products between men and women. Moreover, Milcarz et al. showed that traditional cigarette smokers compared to non-smokers (*p* = 0.05) reported menthol cigarettes to be less harmful than traditional cigarettes [22]. This observation is in line with our study. In the study by Kaleta et al. carried out among young people from a socio-economically disadvantaged rural area in Poland, students perceived slim cigarettes and menthol cigarettes as less harmful [23]. In our study, there were no differences in perception of slim and menthol cigarettes between the age groups. In the study by Majek et al. 95% of medical students believed that HTPs are harmful to health [24]. In our study, 22% of respondents declared that HTPs are less harmful than traditional cigarettes. Differences between the abovementioned studies and our study may result from different populations (general population in our study vs. specific populations (adolescents, medical students, social care beneficiaries) in other studies).

This study has practical implications for tobacco control policy. First, our findings suggest that there is an urgent need to conduct a nationwide information campaign on the cardiovascular effect of tobacco use. Secondly, significant differences in the perception of harmfulness of various tobacco products and e-cigarettes and awareness of the health effects of using tobacco and e-cigarettes between smokers and non-smokers point to the need to implement personalized communication targeted to smokers. Thirdly, our findings indicate a further need for legislative action and tightening the tobacco control Act, especially in terms of protection against secondhand smoke and ban on tobacco advertising, promotion, and sponsorship.

This study has several limitations. First, this study was carried out in a nationwide representative sample of Poles aged 15+ years. It is known that some groups (lower educated, unemployed) may be more vulnerable to use tobacco products and maybe an attractive target group for the tobacco companies [32]. Due to the study design, we cannot comprehensively assess the attitudes towards various tobacco products in this group. Secondly, the perception of the harmfulness of various tobacco products and e-cigarettes was assessed relative to traditional cigarettes. The aggressive marketing of novel nicotine-containing products may influence the perception of individual products, so in this single-point study, we cannot assess changes in attitudes towards various products. However, the same approach was applied in numerous studies on harm perception of tobacco products [18,19,20]. Thirdly, smoking status was defined based on self-reported data on smoking behaviors, so we cannot exclude the possibility of recall bias. The smoking status was not verified with biomarkers of tobacco exposure [33]. Moreover, the measure of harm perceptions is based on a single item per tobacco product and may affect the reliability of the measure. Further studies should focus on face-to-face interviews, quantitative studies as well as the assessment of the perception of different nicotine-containing products, including the perception of advertisements for these products, design, packaging to better characterize the social attitudes towards these products.

## 5. Conclusions

This study demonstrated that among inhabitants of Poland aged 15 years and over, non-combustible tobacco products and e-cigarettes are perceived as less harmful than combustible tobacco products. The current study indicated significant gaps in awareness of the cardiovascular effects of smoking among inhabitants of Poland. Smokers compared to non-smokers were less likely to declare that smoking causes stroke or myocardial infarction. Further legislative actions are needed to adapt the tobacco control act in Poland to the current challenges of tobacco control policy resulting from new nicotine-containing products and their promotion by the tobacco industry.

## Figures and Tables

**Table 1:** Awareness of the health effects of using tobacco and e-cigarettes by smoking status (*n* = 1011).

Characteristics	Total *n* = 1011	Smokers *n* = 226	Non-Smokers *n* = 785	*p*
	Weighted % (95%CI)	Weighted % (95%CI)	Weighted % (95%CI)	
Do you think that smoking causes serious diseases?				
Yes	83.6 (81.2–85.7)	80.1 (74.4–84.8)	84.6 (81.9–86.9)	0.1
No	10.6 (8.8–12.6)	11.5 (8.0–16.3)	10.3 (8.4–12.6)	
I do not know	5.8 (4.6–7.5)	8.4 (5.5–12.8)	5.1 (3.8–6.9)	
Do you think that smoking causes stroke?				
Yes	70.4 (67.5–73.2)	56.6 (50.1–62.9)	74.4 (71.2–77.3)	<0.001
No	15.7 (13.6–18.1)	26.6 (21.2–32.7)	12.6 (10.5–15.1)	
I do not know	13.9 (11.9–16.1)	16.8 (12.5–22.2)	13.0 (10.8–15.5)	
Do you think that smoking causes myocardial infarction?				
Yes	81.4 (78.9–83.7)	70.8 (64.6–76.3)	84.5 (81.8–86.8)	<0.001
No	10.3 (8.6–12.3)	16.8 (12.5–22.2)	8.4 (6.7–10.6)	
I do not know	8.3 (6.8–10.2)	12.4 (8.7–17.3)	7.1 (5.5–9.2)	
Do you think that smoking causes lung cancer?				
Yes	88.9 (86.8–90.7)	87.2 (82.2–90.9)	89.4 (87.1–91.4)	0.5
No	7.5 (6.1–9.3)	8.0 (5.1–12.2)	7.4 (5.8–9.4)	
I do not know	3.6 (2.6–4.9)	4.8 (2.7–8.5)	3.2 (2.2–4.7)	
Do you think that e-cigarette use causes serious diseases?				
Yes	57.9 (54.8–60.9)	49.1 (42.7–55.6)	60.4 (56.9–63.8)	0.01
No	14.3 (12.2–16.5)	16.4 (12.1–21.8)	13.7 (11.4–16.2)	
I do not know	27.8 (25.1–30.6)	34.5 (28.6–40.9)	25.9 (22.9–29.0)	
Do you think that e-cigarette use causes stroke?				
Yes	54.4 (51.3–57.5)	41.0 (34.8–47.5)	58.2 (54.7–61.6)	<0.001
No	15.9 (13.8–18.3)	20.7 (15.9–26.4)	14.5 (12.2–17.2)	
I do not know	29.7 (27.0–32.7)	38.3 (32.3–44.8)	27.3 (24.3–30.5)	
Do you think that e-cigarette use causes myocardial infarction?				
Yes	59.4 (56.3–62.3)	45.1 (38.8–51.7)	63.4 (60.0–66.7)	<0.001
No	12.5 (10.7–14.8)	18.2 (13.7–23.7)	11.0 (9.0–13.3)	
I do not know	28.1 (25.4–30.9)	36.7 (30.7–43.2)	25.6 (22.7–28.8)	
Do you think that e-cigarette use causes lung cancer?				
Yes	65.8 (62.8–68.6)	57.1 (50.6–63.4)	68.3 (64.9–71.4)	0.01
No	11.1 (9.3–13.2)	14.6 (10.6–19.8)	10.0 (8.2–12.4)	
I do not know	23.1 (20.7–25.8)	28.3 (22.9–34.5)	21.7 (18.9–24.7)	
Are you concerned about the harmful effect of passive smoking on your health?				
I am very concerned	9.2 (7.6–11.1)	6.2 (3.7–10.1)	10.0 (8.2–12.4)	<0.001
I am a little concerned	38.4 (35.4–41.4)	58.8 (52.3–65.1)	32.5 (29.3–35.8)	
I am not very concerned	26.6 (24.0–29.4)	25.7 (20.4–31.7)	26.9 (23.9–30.1)	
I am completely not concerned	25.8 (23.2–28.6)	9.3 (6.2–13.8)	30.6 (27.5–33.9)	

**Table 2:** Harm perceptions of various tobacco products and e-cigarettes by smoking status (*n* = 1011).

Harmfulness of Selected Products		Menthol Cigarettes			Flavored Cigarettes (Except Menthol)			Slim Cigarettes			Cigarillos		
		Less Harmful	As Harmful	More Harmful	Less Harmful	As Harmful	More Harmful	Less Harmful	As Harmful	More Harmful	Less Harmful	As Harmful	More Harmful
Overall	% *	15.6	80.4	4.0	16.1	80.0	3.9	17.1	80.0	2.9	12.6	83.1	4.3
*n* = 1011	95%CI	13.5–18.0	77.9–82.7	2.9–5.3	14.0–18.5	77.3–82.3	2.8–5.2	14.9–19.6	77.4–82.4	2.0–4.1	10.7–14.8	80.7–85.3	3.2–5.7
Smokers	% *	19.9	73.9	6.2	17.8	76.0	6.2	20.8	76.1	3.1	13.8	79.6	6.6
*n* = 226	95%CI	15.2–25.6	67.8–79.2	3.7–10.1	13.3–23.3	70.0–81.1	3.7–10.2	16.0–26.6	70.1–81.2	1.5–6.3	9.9–18.9	73.8–84.3	4.1–10.7
Non-smokers	% *	14.4	82.3	3.3	15.7	81.1	3.2	16.1	81.1	2.8	12.2	84.2	3.6
*n* = 785	95%CI	12.1–17.0	79.5–84.8	2.3–4.8	13.3–18.4	78.3–83.7	2.2–4.7	13.7–18.8	78.3–83.7	1.9–4.2	10.1–14.7	81.5–86.6	2.5–5.1
*p*		0.01			0.07			0.2			0.1		
**Harmfulness of Selected Products**		**Smokeless Tobacco**			**Water Pipe (shisha)**			**E-Cigarettes**			**Heated Tobacco Products**		
		**Less** **Harmful**	**As** **Harmful**	**More** **Harmful**	**Less** **Harmful**	**As** **Harmful**	**More** **Harmful**	**Less** **Harmful**	**As** **Harmful**	**More** **Harmful**	**Less** **Harmful**	**As** **Harmful**	**More** **Harmful**
Overall	% *	25.0	71.7	3.3	24.5	71.1	4.4	21.6	70.0	8.4	22.0	71.8	6.2
*n* = 1011	95%CI	22.5–27.8	68.8–74.4	2.3–4.6	22.0–27.3	68.2–73.8	3.3–5.9	19.1–24.2	67.1–72.8	6.9–10.3	19.5–24.6	69.0–74.5	4.9–7.9
Smokers	% *	32.3	63.7	4.0	32.3	62.0	5.7	28.6	61.7	9.7	24.8	66.8	8.4
*n* = 226	95%CI	26.5–38.7	57.3–69.7	2.1–7.4	26.5–38.7	55.5–68.0	3.4–9.6	23.2–34.8	55.2–67.8	6.5–14.2	19.6–30.8	60.4–72.6	5.5–12.8
Non-smokers	% *	22.9	74.0	3.1	22.3	73.6	4.1	19.5	72.4	8.1	21.2	73.3	5.5
*n* = 785	95%CI	20.2–26.0	70.8–76.9	2.1–4.5	19.5–25.3	70.5–76.6	2.9–5.7	16.9–22.4	69.1–75.4	6.3–10.1	18.4–24.1	70.0–76.2	4.2–7.4
*p*		0.01			0.003			0.01			0.1		

*p*—smokers vs. non-smokers; * demographic weighting was applied.

**Table 3:** Awareness of selected health effects of using tobacco and e-cigarettes by sociodemographic factors: Odds ratios (OR) and 95% confidence intervals (95%CI), *n* = 1011.

Variable	Smoking Causes Stroke		Smoking Causes Myocardial Infarction		E-Cigarette Use Causes Serious Diseases	
	Univariate Logistic Regression	Multivariate Logistic Regression	Univariate Logistic Regression	Multivariate Logistic Regression	Univariate Logistic Regression	Multivariate Logistic Regression
	OR (95%CI)	OR (95%CI)	OR (95%CI)	OR (95%CI)	OR (95%CI)	OR (95%CI)
Smoking status						
smoker	0.45 (0.33–0.61) **	0.43 (0.31–0.59) **	0.44 (0.31–0.62) **	0.41 (0.29–0.60) **	0.64 (0.47–0.86) *	0.63 (0.46–0.86) *
non-smoker	Reference	Reference	Reference	Reference	Reference	Reference
Gender						
female	Reference	Reference	Reference	Reference	Reference	Reference
male	0.92 (0.71–1.21)	1.00 (0.75–1.33)	0.95 (0.69–1.30)	1.13 (0.81–1.59)	0.91 (0.71–1.17)	0.95 (0.73–1.23)
Age (years)						
15–19	0.75 (0.42–1.33)	0.56 (0.30–r1.05)	0.52 (0.28–0.99) *	0.35 (0.17–0.70) *	0.93 (0.55–1.61)	0.93 (0.52–1.68)
20–29	1.19 (0.77–1.84)	1.18 (0.73–1.93)	0.95 (0.57–1.58)	0.93 (0.52–1.66)	0.88 (0.60–1.30)	0.76 (0.49–1.18)
30–39	1.02 (0.68–1.53)	1.17 (0.72–1.91)	0.84 (0.52–1.35)	0.93 (0.53–1.66)	1.22 (0.84–1.78)	1.15 (0.74–1.80)
40–49	1.05 (0.68–1.63)	1.22 (0.73–2.04)	0.84 (0.50–1.41)	1.03 (0.56–1.88)	1.28 (0.85–1.93)	1.25 (0.78–2.01)
50–59	0.89 (0.59–1.36)	1.02 (0.63–1.65)	0.93 (0.56–1.55)	1.15 (0.64–2.07)	1.10 (0.75–1.63)	1.08 (0.69–1.68)
60+	Reference	Reference	Reference	Reference	Reference	Reference
Educational level						
primary	0.87 (0.57–1.32)	1.10 (0.65–1.85)	0.72 (0.43–1.20)	1.13 (0.60–2.12)	0.69 (0.47–1.01)	0.90 (0.56–1.45)
vocational	0.79 (0.54–1.18)	0.92 (0.59–1.44)	0.52 (0.33–0.84) *	0.67 (0.39–1.14)	0.70 (0.49–1.01)	0.88 (0.58–1.33)
secondary	1.05 (0.72–1.53)	1.10 (0.74–1.65)	0.82 (0.51–1.31)	0.92 (0.56–1.52)	1.07 (0.75–1.51)	1.26 (0.87–1.82)
higher	Reference	Reference	Reference	Reference	Reference	Reference
Occupational status						
active	Reference	Reference	Reference	Reference	Reference	Reference
passive	0.95 (0.72–1.26)	1.09 (0.73–1.63)	1.02 (0.73–1.41)	1.30 (0.82–2.08)	0.83 (0.64–1.07)	0.99 (0.69–1.42)
Financial situation						
bad	0.84 (0.58–1.23)	0.90 (0.59–1.38)	0.46 (0.28–0.74) **	0.48 (0.28–0.81) *	0.61 (0.43–0.88) *	0.71 (0.48–1.05)
moderate	1.09 (0.78–1.54)	1.21 (0.83–1.76)	0.58 (0.37–0.92) *	0.66 (0.41–1.08)	0.66 (0.47–0.91) *	0.73 (0.51–1.03)
good	Reference	Reference	Reference	Reference	Reference	Reference
Place of residence						
rural	1.12 (0.85–1.48)	1.09 (0.82–1.45)	0.76 (0.55–1.04)	0.77 (0.55–1.08)	0.86 (0.66–1.10)	0.87 (0.67–1.14)
urban	Reference	Reference	Reference	Reference	Reference	Reference

** *p* < 0.001; * *p* < 0.01.

## Data Availability

Data are available on reasonable request. The dataset used to conduct the analyses is available from corresponding author on reasonable request.
